# Esthetic Crown Lengthening and Minimally Invasive Laminate Veneers to Resolve Altered Passive Eruption

**DOI:** 10.1155/2024/8882326

**Published:** 2024-05-24

**Authors:** Cimara Fortes Ferreira, Edival Barreto Magalhães, Barbara Zini

**Affiliations:** ^1^ Department of Periodontology University of Tennessee Health Sciences College of Dentistry, Memphis, TN, USA; ^2^ Private Practice, Florianópolis, SC, Brazil

## Abstract

**Background:**

Altered passive eruption (APE) in the esthetic zone can be an esthetic concern to the patient. To restore adequate crown dimensions, crown lengthening procedures may be necessary.

**Methods:**

The present case is a report of a patient with an unsatisfied smile due to a complaint of short clinical crowns. The periodontal diagnosis was APE and deviated maxillary anterior midline. A mock-up was made to assist the provider in evaluating the patient's esthetic concerns and acceptance of the proposed treatment plan.

**Results:**

Esthetic crown lengthening and ultrathin ceramic laminate veneers were used to treat an APE type I subdivision B, resulting in a gingival display reduction and in final PES/WES scores of 10.

**Conclusion:**

The use of periodontal plastic surgery in conjunction with ultrathin ceramic laminate veneers was mandatory to restore an unesthetic smile. The proposed treatment reduced the gingival display significantly and increased the crown height to length proportions reaching an esthetic smile and patient satisfaction.

## 1. Introduction

The esthetic appearance of an individual's smile plays a significant role in their social and psychological well-being [1]. A “gummy smile,” characterized by an excessive display of gingival tissues (>3 mm) during smiling, is considered unattractive [2, 3]. A pleasant smile is when the gingival margin of the maxillary teeth is approximately 1 mm from the upper lip. To maintain a pleasant smile, it is recommended that this distance does not exceed 2-3 mm.

Several factors, including altered passive eruption (APE) [4], vertical maxillary excess [5], and hypermobile upper lip [6], can contribute to this condition.

APE, a localized tooth-related factor classified as developmental or acquired deformity and condition [7], is subdivided in two types. Type I involves the gingival margin being incisal or occlusal to the cementoenamel junction (CEJ), with the mucogingival junction (MGJ) positioned apical to the crest of the bone. Type II is characterized by a normal gingival dimension, with the free gingival margin incisal or occlusal to the CEJ and the MGJ positioned at the CEJ. Both types have subdivisions, with subdivision A indicating that the alveolar crest is 1.5 to 2 mm apical to the CEJ and subdivision B indicating that the alveolar crest is coincident with the CEJ [8].

Treatment options for APE type I include gingivectomy, apically positioned flap, and osseous resective surgery. A comprehensive treatment plan involving prosthodontics, orthodontics, and periodontics is necessary for addressing APE type II. However, caution must be exercised during osseous resective surgeries to prevent excessive bone resection and subsequent gingival recession, which can lead to esthetic complications [9].

The present case report is a Coslet [8] type I subdivision B that required surgical crown lengthening using selective osseous correction [10].

## 2. Case Description and Results

The patient, a 46-year-old Caucasian female, presented to a private clinic with a chief complaint of unpleasant esthetics. Her medical history was noncontributory. A clinical evaluation was conducted at the first appointment following the clinical protocol described in the literature [11]. Her dental history revealed significant dental restorative work that had been done in the last 16 years. Her periodontal probing depths were within 1-3 mm. She was diagnosed with periodontal health and with a developmental and acquired condition [7], APE [12], and Coslet type I subdivision B [8]. She presented facial and lip symmetry and normal lip mobility. The maxillary anterior teeth showed normal width but reduced length, and she showed a slight right deviation of her maxillary anterior midline.

**Figure 1:**
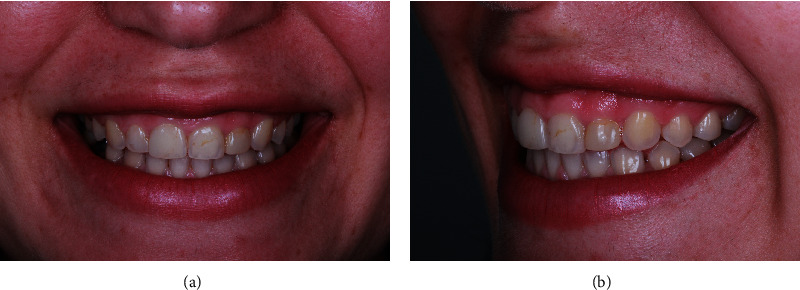
(a) Frontal view. Note the excessive gingival display and reduced height for the anterior maxillary teeth during patient's high smile. (b) Perspective view. Note the reduced size of the patient's #9 and 10.

An intraoral evaluation revealed that the position of the mucogingival junction is approximately 5 mm from the gingival sulcus, characterizing excessive gingival display [4] (Figure 1).

**Figure 2:**
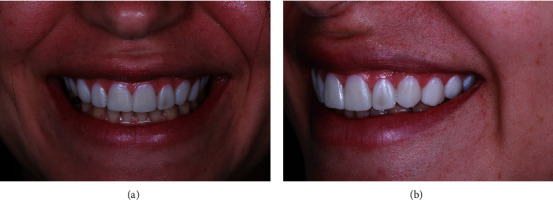
(a) Frontal and (b) perspective views of the patient's smile using the mock-up. Note an esthetically pleasing smile.

The use of a digital workflow in dentistry has been detailed as a step-by-step process to enhance the gingival architecture in the esthetic zone [13]. The patient's dental casts were created, and a mock-up was developed using the Digital Smile Design® protocol [14] to achieve an esthetically pleasant dental arrangement that harmonizes with the patient's facial features [15] (Figure 2). The patient expressed satisfaction with the proposed esthetic solution and approved the treatment plan. The initial assessment using the pink esthetic score (PES) [16] indicated scores ranging from 8 to 9 for the 6 anterior maxillary teeth, primarily due to tooth contour. The white esthetic score (WES) [16] for the same teeth varied from 3 to 5, mainly attributed to the lack or absence of tooth form, volume, color surface texture, and translucency. The treatment plan included crown lengthening and 0.4-0.6 mm wide ultrathin ceramic laminates, as well as lithium disilicate laminate veneers (LDLV) from teeth #5-13. The successful use of layered pressed-ceramic LDLV technique has been described in the literature [17]. The patient approved the treatment plan, and in the following week, the crown lengthening was conducted.

**Figure 3:**
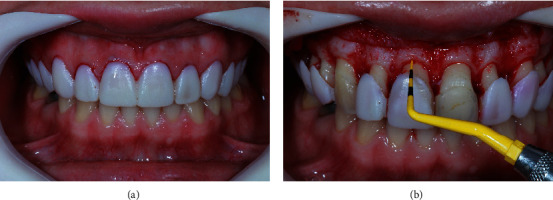
(a) Mock-up used as esthetic stent for the crown lengthening procedure planned for teeth #4-14. (b) A periodontal probe was used to assist during the bone reduction.

**Figure 4:**
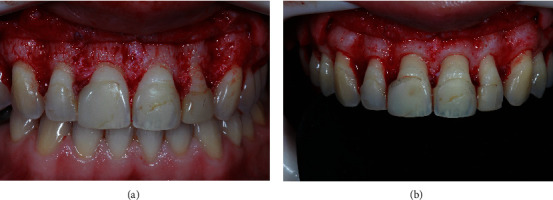
(a) Frontal view after a full-thickness flap was elevated. Note the presence of the bone at the level of the cementoenamel junction from teeth #6-11. (b) Frontal view of the bone after bone reduction was conducted. Note the presence of extensive overhang of the mesial aspect of the restorations for tooth #8.

The surgical appointment involved using the mock-up as an esthetic stent for the crown lengthening procedure (Figure 2). After administering anesthesia, the esthetic stent was inserted, and the future position of the CEJ was marked. An internal bevel incision was made based on the markings for each tooth, preserving the interdental papilla. Subsequently, an intrasulcular internal bevel incision (Swan Morton, UK) was made with a 15C blade (Figure 3(a)), and a collar was obtained. After collar removal, subgingival enamel was exposed. The golden proportions were rechecked using a periodontal probe (Figure 3(b)). Next, a full-thickness buccal flap was elevated to the level of the mucogingival junction. After flap reflection, the bone showed to be at the CEJ (Figure 4(a)). The distance from the bone crest to the CEJ was measured transsurgically, using the esthetically driven surgical stent (mock-up), for the osteotomy. The osteotomies were performed to achieve 2 mm between the CEJ of the crown line angles and the esthetic gingival stent margins. A 1-milimeter bone reduction was carried out from the line angles to the mesial and distal proximal sites of the proposed restorative margins. No bone reduction was performed interproximally for the mesial sites of the central incisors. A periodontal probe was used to assist the bone reduction procedure. Manual (#2 Fedi, #36/37 Rhodes chisels) and rotatory instruments were used for osteotomy and osteoplasty. Following bone reduction (Figure 4(b)), the surgical sites were thoroughly irrigated with saline solution, and the buccal flap was repositioned. Next, digital compression was conducted for 1 minute, and a 3/8 circle 13 mm needle with a 6-0 polypropylene thread (Atramat, Japan) was used to stabilize the flap with simple interrupted sutures. The patient received postoperative instructions and was placed on a pain control regimen (750 mg of paracetamol qid for the following 3 days). Oral hygiene instructions, including the use of 0.12% chlorhexidine gluconate oral rinse (PerioGard®, Colgate, Johnson & Johnson) twice daily for 2 weeks, were provided, and the patient was advised to refrain from mechanical plaque control in the operated sextants for 2 weeks. Additionally, the patient was instructed to refrain from toothbrushing for 2 weeks, apply ice packs for the first 24 hrs. postsurgically, consume only soft foods during the first week, and avoid any other mechanical trauma to the surgical sites. Flossing was permitted for the mesial aspect of the central maxillary incisors after 10 days postoperatively and 21 days PO otherwise, to allow sufficient healing of the interproximal sites that received a bone reduction procedure.

After 7 days, the patient was instructed to use a 2-year-old soft bristle pediatric toothbrush, brushing only in the direction from the gingival tissues towards the tooth. The patient was discharged, and the sutures were removed at the 10-day appointment.

**Figure 5:**
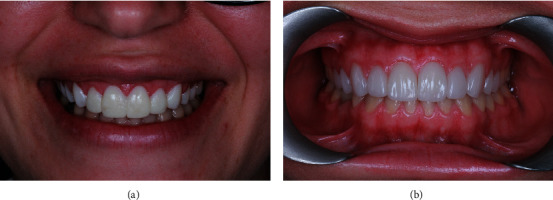
(a) Fifteen days postoperatively after the sutures were removed and the provisional restorations were placed. The patient returned for suture removal. Note the presence of slight marginal erythema, which may be expected at this follow-up period. (b) Frontal view 6 months postoperatively with the final laminate veneers.

At the 15-day postoperative appointment, the patient received prophylaxis prior to suture removal (Figure 5(a)). The provisional restorations were placed after 60 days of healing. The provisional restorations were placed after 90 days of healing, and the definitive restorations were cemented 6 months postoperatively using a photopolymerizing resin-luting cement (Variolink Esthetic, Ivoclar Vivadent) (Figure 5(b)).

**Figure 6:**
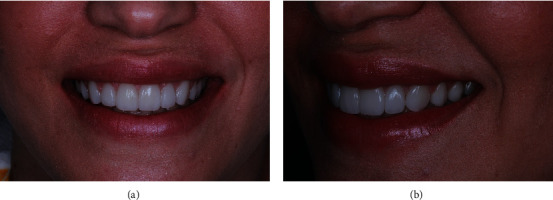
(a) Frontal view of the patient's smile six months postoperatively. Note the esthetic smile. (b) Perspective view of the patient's smile 6 months postoperatively.

During the six-month follow-up, the patient exhibited an esthetic smile (Figures 6(a) and 6(b)), attributed to achieving esthetic equilibrium through adhering to the golden proportion measurements for the anterior maxillary teeth. The patient's final PES [16] was 10 for the 6 anterior maxillary teeth due to recovery of the gingival contours. For the same teeth, the patient's final WES [16] was 10 due to complete tooth form, volume, color surface texture, and translucency.

**Figure 7:**
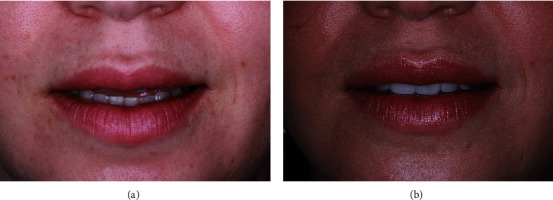
(a) Preoperative frontal view of the lack of lip in repose. (b) Postoperative frontal view of the lip in repose showing the presence incisal edges of the anterior maxillary teeth.

The six-month follow-up showed significant esthetic smile and stability of the tissues (Figures 6(a) and 6(b)). The patient also regained esthetic lip support before (Figure 7(a)) after the rehabilitation was completed (Figure 7(b)).

## 3. Discussion

The altered passive eruption is commonly addressed with an esthetic crown lengthening procedure, which involves gingivectomy or apically positioned flap with or without ostectomy [10]. Esthetic crown lengthening procedures have been documented in a controlled clinical trial [18]. In the present case, the patient exhibited gingival display of 3 mm or more when smiling, a finding known to impact esthetics negatively [19]. The proposed treatment of crown lengthening and LDLV successfully addresses the patient's esthetic concern. The concept of patient satisfaction has been evaluated in dentistry and medicine revealing its multidimensional characteristic and the need for a better definition [20]. The present treatment combined esthetic crown lengthening, using the Digital Smile Design (DSD) concept, and a digital wax-up to fabricate pressed LDLV layered with feldspathic porcelain [17].

In addition, Belser et al.'s proposed modification to the PES and the WES [16] was used to evaluate the esthetics before and after treatment. This method of evaluation quantifies esthetics, allowing for comparison of treatment results between studies. The proposed treatment of an APE type I subdivision B, with esthetic crown lengthening and the use of ultrathin LDLV, resulted in an increase in the PES from 8 to 10 and a significant increase in WES from 3-5 to 10. These scores quantify the deficient patient; however, the “gummy smile,” which would be represented by the pink esthetics, showed to be of lower importance when using the scale when compared to the WES, which was significantly low at the patient's initial visit. The authors suggest a limitation of the PES score for “gummy smile” cases. Adding the length of the gingival tissues to this evaluation would facilitate giving them a more accurate esthetic score.

Mootha et al. [21] compared the use of different tools for esthetic treatment planning the anterior maxillary teeth and their relation to the various geometric proportions in an Indian population sample. In this study, the use of the DSD® software protocol and Chu's proportion gauge [22] range leads to pleasing smiles in the studied population. The present case report used the DSD® protocol for the esthetic treatment planning of the anterior maxillary teeth.

The literature supports the use of digital workflow to improve treatment planning for gingival/tooth architecture in the esthetic zone. The use of a diagnostic mock-up or overlay as a crown lengthening surgical guide to improve the “gummy smile” has shown to be a viable option as a surgical guide for crown lengthening [13]. The present case report used a digitally made esthetic stent to guide the surgeon during the surgical procedure, and the patient accepted outcomes presented during the treatment planning phase.

This cement system used in the present case report utilizes a novel dibenzoyl germanium derivative photoinitiator which exhibited statistically superior color stability and a higher degree of conversion when compared to Calibra, Variolink-N, and NX3 resin cements in an *in vitro* setting [23].

A limitation of this case report is the lack of use of digital technology to precisely measure the amount of increased lip support reached with the executed treatment.

## 4. Conclusion

In the present case report, an APE type I subdivision B case was treated with esthetic crown lengthening and minimally invasive LDLV to resolve the patient's esthetic concern. The proposed treatment reduced the gingival display significantly and increased the crown height to length proportions reaching an esthetic smile and patient satisfaction.

## Additional Points

*Clinical Relevance.* A gummy smile can result in reduced self-esteem. There are different ways to treat this condition. This case report describes a step-by-step multidisciplinary approach in treatment planning for esthetic crown lengthening in conjunction with minimally invasive laminate veneers to treat the gummy smile, offering valuable insights for clinicians dealing with similar esthetic challenges in their practice.
